# Antibodies to NCP, RBD and S2 SARS-CoV-2 in Vaccinated and Unvaccinated Healthcare Workers

**DOI:** 10.3390/vaccines10081169

**Published:** 2022-07-22

**Authors:** Agata Błaszczuk, Aleksander Michalski, Maria Malm, Bartłomiej Drop, Małgorzata Polz-Dacewicz

**Affiliations:** 1 Department of Virology with SARS Laboratory, Medical University of Lublin, 20-093 Lublin, Poland; m.polz@umlub.pl; 2 1st Clinical Military Hospital with Outpatient Clinic in Lublin, 20-049 Lublin, Poland; aleksander.michalski@1wszk.pl; 3 Department of Computer Science and Medical Statistics with the e-Health Laboratory, 20-090 Lublin, Poland; maria.malm@umlub.pl (M.M.); bartlomiej.drop@umlub.pl (B.D.)

**Keywords:** COVID-19, HCWs, SARS-CoV-2 antibody, NCP, RBD, S2, vaccination

## Abstract

In a few months, the SARS-CoV-2 virus caused a worldwide COVID-19 pandemic. In Poland, 6 million cases of the disease and 113,000 deaths from COVID-19 have been reported. Healthcare workers (HCWs) constitute one of the main COVID-19 risk groups. The Microblot-Array COVID-19 IgG assay was used to detect antibodies against three major SARS-CoV-2 antigens: nucleocapsid (NCP), RBD, and Spike 2 (S2). The aim of our study was to determine the seroprevalence and titer of anti-SARS-CoV-2 IgG antibodies—NCP, RBD, and S2—as markers of the humoral response in vaccinated and unvaccinated HCWs. The study included 203 persons who were divided into four groups: “COVID-19 Vaccinated”, “COVID-19 Unvaccinated”, “Non-COVID-19 Vaccinated”, and “Non-COVID-19 Unvaccinated”. The obtained results indicate that both seroprevalence and the antibody titer are the highest in the “COVID-19 Vaccinated” group. There is no so-called sterile vaccination, and after 6 months from the second dose of vaccine, most vaccinated people have a fairly high level of antibodies. We suggest that multiple vaccination and continuous testing are necessary. The Microblot-Array assay can distinguish between antibodies acquired after infection and/or vaccination.

## 1. Introduction

SARS-CoV-2, an etiological agent of COVID-19, has affected millions of people worldwide, causing the largest global pandemic in recent history. Up until 15 May 2022, over 518 million cases and over 6 million deaths have been reported all over the world, and in Poland, 6 million cases and 113,000 deaths have been reported [1]. Healthcare workers (HCWs) belong to the high-risk group for COVID-19 infection. High morbidity and mortality due to COVID-19 caused many laboratories in the world to begin intensive research on the development of an effective vaccine. The vaccination campaign was started in Europe in December 2020. The first vaccine, accepted by the European Medicines Agency (EMA) and made available for immunization in Poland, was the BNT162b2 mRNA COVID-19 vaccine (Comirnaty; BNT162b2, BioN-Tech/Pfizer, Mainz, Germany/New York, NY, USA). The active substance in Pfizer-BioNTech Comirnaty vaccine is the mRNA encoding the S protein of SARS-CoV-2 [2]. The effective immunization of HCWs plays a key role in preventing the spread of SARS-CoV-2 infection by evoking the production of virus-specific neutralizing antibodies (NAbs) and cellular immunity (CD4+ and CD8+ cells, with IFN playing a pivotal role) [3,4,5]. Previous studies have shown the high efficacy and safety of the vaccine [6,7].

There are four major structural proteins in SARS-CoV-2: spike (S), envelope (E), membrane (M), and nucleocapsid (NCP) encoded by the S, E, M, and N genes, respectively [8,9]. The S protein contains the S1 and S2 subunits. The RBD in S1 binds to angiotensin-converting enzyme 2 (ACE2) on the host cell, changing the Spike 2 (S2) subunit conformation and viral particle entry through virus–host cell membrane fusion. Antibodies against all major viral antigens are detectable both during or after COVID-19, as well as after vaccination [10].

The aim of this study was to evaluate the seroprevalence and the level of anti-SARS-CoV-2 IgG antibodies against the NCP, RBD, and subunit S2 of the virus’s S protein as a marker of the humoral response in vaccinated and unvaccinated HCWs. Persons vaccinated with two doses of the vaccine and those who, despite vaccination, underwent SARS-CoV-2 infection, as well as those who were neither sick nor vaccinated, were included. The incidence of antibodies and their level according to age and sex of respondents was also analyzed.

## 2. Materials and Methods

### 2.1. Study Design

The present study was conducted between November 2021 and December 2021. A total of 350 medical staff from Clinic Hospital in Lublin participated voluntarily in the study. People with chronic diseases (respiratory, endocrine, and cardiovascular diseases; diabetes; allergies; cancer) and working in COVID-19 wards were excluded from the study. The study participants also included people vaccinated with two doses of Pfizer vaccine (the second dose of the vaccine had to have been administered no later than 6 months before the study) and those who had not been vaccinated against COVID-19 at all. People who had a mild history of SARS-CoV-2 infection and did not require hospitalization and those who reported in the interview that they had not been infected with COVID-19 were qualified. The COVID-19 infection was confirmed by a documented positive RT-PCR test using a nasopharyngeal swab.

Finally, 203 people were qualified for our study and were divided into 4 groups:

1. vaccinated individuals undergoing COVID-19 infection (“COVID-19 Vaccinated”);
2. unvaccinated individuals undergoing COVID-19 infection (“COVID-19 Unvaccinated”);
3. vaccinated individuals who did not have COVID-19 infection (“Non-COVID-19 Vaccinated”);
4. unvaccinated individuals who did not have COVID-19 infection (“Non-COVID-19 Unvaccinated”).

All participants answered a questionnaire with demographic, epidemiological, and clinical information and regarding previous exposure to COVID-19.

### 2.2. Sample Collection

Venous blood samples (3–5 mL) from all subjects were centrifuged at 1500× *g* rpm/15 min at room temperature, and then the serum was stored at 4 °C and used within five days.

### 2.3. Detection of SARS-CoV-2 Antibody

The serum samples from all individuals were tested using the Microblot-Array COVID-19 IgG assay (TestLine Clinical Diagnostics, Brno, Czech Republic) according to the manufacturer’s instructions. This test detects the presence and titer of specific anti-SARS-CoV-2 antibodies, NCP, RBD, S2, E, papain-like protease (PLpro), and ACE2. In addition, this test detects cross-reactivity with other coronaviruses such as Middle East respiratory syndrome coronavirus (MERS-CoV), severe acute respiratory syndrome coronavirus (SARS-CoV), human coronavirus 229E (HCoV 229E), and human coronavirus NL63 (HCoV NL63). The results are given in units of U/mL. The interpretation takes into account the presence or absence of a reaction against at least 1 antigen—NCP, RBD, or S2.

The results were interpreted as follows: <185 U/mL = negative, 185–210 U/mL = borderline, >210 U/mL = positive.

### 2.4. Statistical Analysis

Categorical variables were expressed as counts and percentages. Prevalence of SARS-CoV-2 antibodies was reported as a percentage. The chi-squared test or two-tailed Fisher’s exact test (when chi-squared test assumptions did not hold due to low expected cell counts), where appropriate, was used to compare antibody test results in different groups.

The Kruskal–Wallis test with post hoc Dunns’ test were used to compare four analyzed groups. Furthermore, Spearman correlations were performed to assess the association of antibody levels with age and sex. A *p*-value of less than 0.05 was considered to show a statistically significant result.

### 2.5. Ethics

The research was approved by the Medical University of Lublin Ethics Committee and is in accordance with the GCP regulations (no. KE-0254/121/2021, 27 May 2021). Written informed consent was obtained from each participant.

## 3. Results

### 3.1. Characteristics of the Studied Population

Details of the characteristics of the studied group of 203 participants are presented in Table 1.

Out of 203 tested HCWs, 82.8% (168 persons) were vaccinated with two doses of the Pfizer vaccine, and 42.4% (86 persons) had a mild COVID-19 infection confirmed by RT-PCR (Table 1). A total of 17.2% (35 persons) did not vaccinate against COVID-19 at all, and 57.6% (117 persons) did not undergo COVID-19 infection. The study included 53.2% of physicians (108 persons) and 46.8% of nurses (95 persons) (Table 1).

### 3.2. Prevalence of NCP, RBD, and S2 Antibodies in Studied Persons

**Figure 1:**
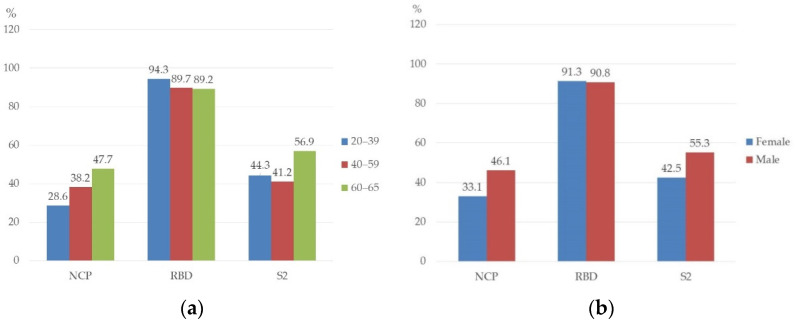
Prevalence of antibodies against SARS-CoV-2 in healthcare workers by age (**a**) and sex (**b**) (%); all groups *p* > 0.05.

The incidence of individual antibodies was also analyzed according to age and sex. There were no statistically significant differences in the antibody frequency according to age and sex (Figure 1). The seroprevalence of RBD antibodies was high in all age ranges—the highest in the age rage 20–39 (94.3%) (Figure 1a). The prevalence of NCP and S2 antibodies were higher in men than in women, 46.1% vs. 33.1% and 55.3% vs. 42.5%, respectively (Figure 1b). However, the differences were not statistically significant.

### 3.3. Prevalence of NCP, RBD, and S2 Antibodies in Studied Groups

Table 2 shows the frequency of antibody occurrence depending on the group. The group “COVID-19 Vaccinated” included 67 persons (33.0%), the group “COVID-19 Unvaccinated”—19 persons (9.4%), the group “Non-COVID-19 Vaccinated”—101 persons (49.7%), and the group “Non-COVID-19 Unvaccinated”—16 persons (7.9%).

NCP, RBD, and S2 antibodies were the most frequently present in persons from the group “COVID-19 Vaccinated”, respectively, 65.7%, 100%, and 85.1%, while in the group “Non-COVID-19 Vaccinated”, the most frequently found antibodies were RBD-97%. In unvaccinated persons who had a mild infection, NCP and S2 antibodies were detected in 42.1% and RBD antibodies in 78.9%. In the serum samples obtained from the “Non-COVID-19 Unvaccinated” population, in four participants (25%), we detected the presence of NCP, RBD, and S2 antibodies (three women and one man) (Table 2). Presumably, these people had an asymptomatic infection. This group was excluded from further analysis.

**Figure 2:**
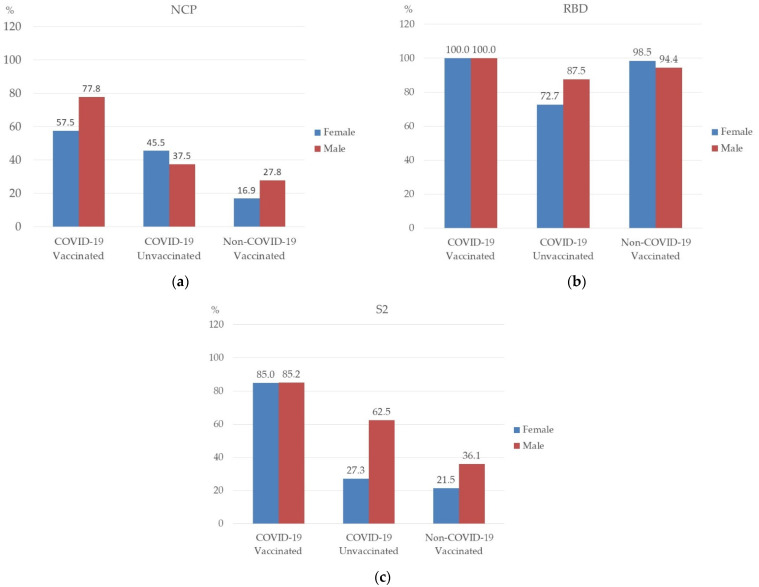
Prevalence of NCP (**a**), RBD (**b**), and S2 (**c**) antibody by sex among the studied groups.

Then, it was analyzed as to whether there were differences in the prevalence of NCP, RBD, and S2 antibodies in each group, depending on sex and age. Statistical analysis showed no significant relationship between sex and the prevalence of antibodies in the studied groups (Figure 2). However, it can be noticed that NCP antibodies in the “COVID-19 Vaccinated” group were more common in men (77.8%) than in women (57.5%) (Figure 2a). The seroprevalence of S2 antibodies in the “COVID-19 Unvaccinated” and “Non-COVID-19 Vaccinated” groups were also higher in men (62.5% and 36.1%, respectively) (Figure 2c). However, as for the prevalence of RBD antibodies in the study groups, they were similar in men and in women (Figure 2b).

**Figure 3:**
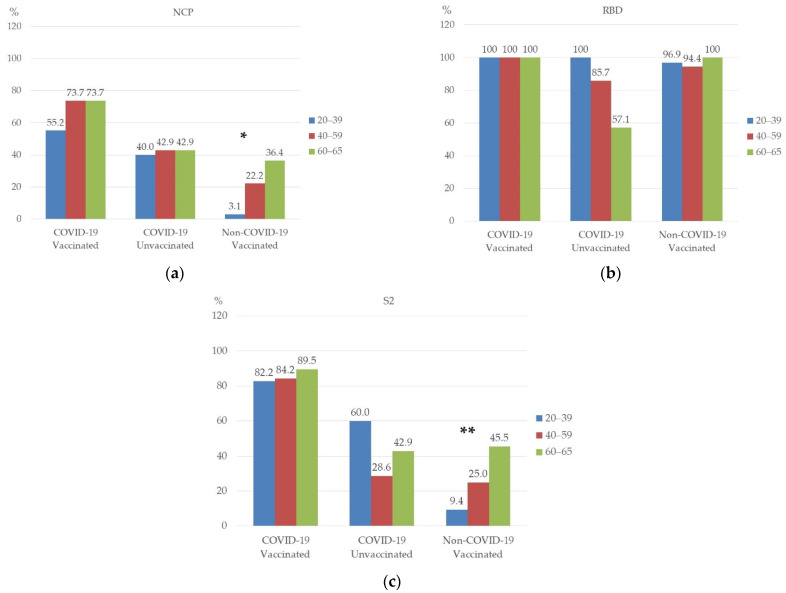
Prevalence of NCP (**a**), RBD (**b**), and S2 (**c**) antibody by age. * *p* = 0.002; ** *p* = 0.005.

Statistical analysis showed a significant relationship between age and the presence of NCP antibodies in the “Non-COVID-19 Vaccinated” group (Figure 3a), *p* = 0.002. NCP antibodies were more common in people aged 60–65.

No significant relationship was found between age and the presence of RBD antibodies in the studied groups (Figure 3b). However, in the “COVID-19 Unvaccinated” group, RBD antibodies were slightly less common in people aged 60–65.

A statistically significant relationship was also found between age and the presence of S2 antibodies in the “Non-COVID-19 Vaccinated” group (Figure 3c), *p* = 0.005. In this group, antibodies were present most often in people aged 60–65.

Another parameter that we compared in this study was the titer of NCP, RBD, and S2 antibodies in each of the studied groups. The highest titer of NCP, RBD, and S2 antibodies was in the “COVID-19 Vaccinated” group (849.2 U/mL, 965.3 U/mL, and 771.6 U/mL, respectively) (Table 3).

No significant differences were found in the level of antibodies in the study groups depending on sex (Table 4). In all tested groups, we were able to observe similar NCP, RBD, and S2 antibody titers in both women and men.

## 4. Discussion

The prevalence of SARS-CoV-2 antibody in medical staff differs between countries and depends on various agents such as study populations, type of antibody test (differences in sensitivity and specificity), and type of data collection. Numerous amounts of research have been carried out to determine humoral immunity. The seroprevalence of IgG antibodies to SARS-CoV-2 was analyzed, both in the general population and in various groups [11,12,13]. Several commercially available serologic tests and various automated analytical platforms have been used in diagnostics for seroprevalence [14,15,16]. The most available tests detect IgG or total antibodies that recognize NCP and/or the S glycoprotein, which are the most significant SARS-CoV-2 antigens [17,18,19].

The virus neutralizing test (VNT) can determine the protective activity of antibodies. However, as demonstrated Montesions et al. [20], there is a significant degree of agreement in the IgG titers measured between Microblot-Array and the VNT. These authors compared the five different serology tests available for identify Nabs SARS-CoV-2 and concluded that Microblot-Array COVID-19 IgG assays could help assess possible waning loss of vaccine protection in the long term. In our study, Microblot-Array COVID-19 IgG was used. In this test, the levels of NCP, RBD, and S2 antibodies were detected.

The meta-analysis revealed the overall seroprevalence of SARS-CoV-2 antibodies among HCWs, with the results ranging from 0% to 45.3%; 8.7% on average [21]. North America demonstrated the highest prevalence of 12.7%, Europe—8.5%, Africa—8.2%, and Asia—4% [22].

One of the first studies on seroprevalence in Poland was the study by Lorent et al. [23], which indicated low herd immunity in the Polish population after the initial wave of the COVID-19 pandemic (the seroprevalence of SARS-CoV-2 antibodies was only 0.93%).

According to our knowledge, the research we have presented is most likely the first one in Poland concerning not only the seroprevalence but also analysis of the titer of antibodies against the three main SARS antigens: NCP, RBD domain, and S2 subunit.

According to Herzberg, healthcare workers achieved a 100% humoral response after two doses of the Pfizer vaccine [24]. In our study, we obtained similar results for the RBD antibodies that appear after vaccination.

NCP, RBD and S2 antibodies were the most frequently found in persons from the group “COVID-19 Vaccinated”, respectively, 65%, 100%, and 85.1%, while RBD in the group “Non-COVID-19 Vaccinated” was detected in 97%. In unvaccinated persons, after undergoing mild infection, the prevalence of NCP and S2 antibodies was 42.1%, and RBD was 78.9%. 

The highest titer of NCP, RBD, and S2 antibodies was in the “COVID-19 Vaccinated” group (849.2 U/mL, 965.3 U/mL, and 771.6 U/mL, respectively).

Sonmezer et al. [25] analyzed the seroprevalence in HCWs before the vaccination program. In asymptomatic individuals, the prevalence of SARS-CoV-2 IgG antibodies in HCWs reached 8.7%, whereas the figure was 1.9% in individuals previously infected with SARS-CoV-2.

Our research showed the presence of IgG antibodies for the virus NCP in 25 persons (including 21 vaccinated and 4 unvaccinated) who did not report COVID-19 history. Therefore, it can be assumed that these people undergone the asymptomatic COVID-19 infection.

Some authors suggest the possibility of nosocomial infections, which may constitute about 15% [26].

In their study, Shields et al. [27] found that people with symptoms of disease had higher seroconversion rates than those with asymptomatic disease. Similarly, our research has shown that in the groups where people have undergone COVID-19, a higher incidence of anti-SARS-CoV-2 antibodies can be seen than in persons without COVID-19 history.

It appears that convalescent persons exhibit a higher titer of antibodies after the first dose of vaccine than those non-infected with SARS-CoV-2 [28].

Miyajima et al. [29] observed that persons who experienced severe fatigue after the second immunization showed a high antibody level that reached the highest result one month after the second dose. A study by Bleier et al. [30] demonstrated the increase in the risk of infection with a simultaneous decrease in the specific antibody level [31].

Currently approved COVID-19 mRNA vaccines generate antibodies to the S protein and not to nucleocapsid proteins that are likely detected only after natural infections. The Microblot-Array test we used allows us to distinguish whether the antibodies were produced as a result of infection or were created after vaccination.

The U.S. Food and Drug Administration (FDA) clearly warns against using currently authorized SARS-CoV-2 antibody tests to evaluate a person’s level of immunity or protection from COVID-19, especially if the person received a COVID-19 vaccination [32].

Lorent et al. [23] states that immune response depends on age. They observed the highest seropositivity in people over 60 years of age. Similarly, in our study, the overall prevalence of NCP and S2 antibodies among HCWs was higher in people aged 60–65. The same relationship can also be seen in the “Non-COVID-19 Vaccinated” group.

Some studies show that antibody levels are similar in men and women [25,33], and other authors observed higher seroprevalence in males than in females [21,34]. Our study did not show any significant differences in the level of antibodies in particular study groups according to sex. Nevertheless, the prevalence of NCP and S2 antibodies was higher in men than in women, at 46.1% vs. 33.1% and 55.3% vs. 42.5%, respectively. In the group “COVID-19 Vaccinated”, the prevalence of NCP antibodies was higher in males (77.8%) than in females (57.5%).

Research by Zeng et al. [35] showed that IgG antibody titer was not affected by sex in the case of mild COVID-19 disease. After suffering from clinically severe COVID-19, high levels of SARS-CoV-2 IgG antibodies in the serum were found in women.

Other authors state that the level of antibodies is higher in younger people [28,35].

Wang et al. [36] found that anti-Spike responses were higher in individuals who received the Pfizer mRNA vaccine, in women, and in younger participants.

The main limitation of our study is the relatively small participants group, especially in terms of non-COVID-19 and unvaccinated individuals. Taking into account the large number of analyzed immunological variables and their complexity, we decided to describe our results. However, more participants would be desirable. Due to the insufficient number of persons in the group “Non-COVID-19 Unvaccinated”, possible age differences in antibody levels were unable to be calculated.

The obtained results indicate that there is no so-called sterile vaccination, and after 6 months from the second dose of vaccine, most vaccinated people have a fairly high level of antibodies. We suggest that multiple vaccination and continuous testing are necessary.

## 5. Conclusions

In conclusion, healthcare workers vaccinated with two doses of COVID-19 mRNA vaccines (BioNTech/Pfizer) have a high antibody titer six months after vaccination. The Microblot-Array assay can distinguish between antibodies acquired after infection and/or vaccination. Future studies are needed to assess the durability of vaccine immunity.

## Figures and Tables

**Table 1:** Characteristics of the study group.

		N	%
**Sex**	Female	127	62.6
	Male	76	37.4
**Age**	20–39	70	34.5
	40–59	68	33.5
	60–65	65	32.0
**COVID-19**	Yes	86	42.4
	No	117	57.6
**Vaccinated**	Yes	168	82.8
	No	35	17.2
**Physicians**		108	53.2
**Nurses**		95	46.8

N—number of participants.

**Table 2:** Prevalence of NCP, RBD, and S2 antibodies in the studied groups.

Participants’ Group	NCP		RBD		S2		Total Participants N = 203	
	N	%	N	%	N	%	N	%
COVID-19 Vaccinated	44	65.7	67	100.0	57	85.1	67	33.0
COVID-19 Unvaccinated	8	42.1	15	78.9	8	42.1	19	9.4
Non-COVID-19 Vaccinated	21	20.8	98	97.0	27	26.7	101	49.7
Non-COVID-19 Unvaccinated	4	25.0	4	25.0	4	25.0	16	7.9

N—number of participants.

**Table 3:** The level of NCP, RBD, and S2 antibodies (U/mL) in the studied groups ^1^.

Participants’ Group	NCP	RBD	S2
COVID-19 Vaccinated	849.2 (399.8–989.2)	965.3 (224.8–976.1)	771.6 (478.3–833.0)
COVID-19 Unvaccinated	559.1 (196.3–969.1)	696.9 (210.0–999.9)	476.1 (187.3–986.1)
Non-COVID-19 Vaccinated	558.7 (196.3–969.1)	888.7 (190.2–1000.5)	472.5 (189.0–1000.0)

^1^ Median (min–max); Mann–Whitney U test. All groups: *p* > 0.05.

**Table 4:** The level of NCP, RBD, and S2 (U/mL) in the studied groups by sex ^1^.

Participants’ Group	NCP		RBD		S2	
	Female	Male	Female	Male	Female	Male
COVID-19 Vaccinated	769.9	928.5	852.8	965.3	774.9	768.2
	(399.8–989.2)	(928.5–928.5)	(224.8–976.1)	(965.3–965.3)	(478.3–833.0)	(768.2–768.2)
COVID-19 Unvaccinated	457.1	623.5	666.8	767.5	474.6	506.7
	(196.3–880.2)	(265.9–969.1)	(227.1–995.6)	(210.0–999.9)	(254.6–797.5)	(187.3–986.1)
Non-COVID-19 Vaccinated	439.8	454.8	871.6	920.5	503.9	464.2
	(210.0–835.1)	(202.2–928.5)	(190.2–1000.5)	(210.0–998.9)	(189.0–1000)	(199.5–995.5)

^1^ Median (min–max); Mann–Whitney U test. All groups: *p* > 0.05.

## Data Availability

The data presented in this study are available in the article.

## References

[1] World Health Organization Weekly Epidemiological Update on COVID-19—18 May 2022. Edition 92. https://www.who.int/publications/m/item/weekly-epidemiological-update-on-COVID-19---18-may-2022.

[2] European Medicines Agency COVID-19 mRNA Vaccine (Comirnaty): EU Summary of Product Characteristics. https://www.ema.europa.eu/en/documents/product-information/comirnaty-epar-product-information_en.pdf.

[3] Sasso B.L., Giglio R., Vidali M., Scazzone C., Bivona G., Gambino C., Ciaccio A., Agnello L., Ciaccio M. (2021). Evaluation of anti-SARS-CoV-2 S-RBD IgG antibodies after COVID-19 mRNA BNT162b2 vaccine. Diagnostics.

[4] Jacofsky D., Jacofsky E.M., Jacofsky M. (2020). Understanding antibody testing for COVID-19. J. Arthroplast..

[5] Sauer K., Harris T. (2020). An effective COVID-19 vaccine needs to engage T cells. Front. Immunol..

[6] Polack F.P., Thomas S.J., Kitchin N., Absalon J., Gurtman A., Lockhart S., Perez J.L., Pérez Marc G., Moreira E.D., Zerbini C. (2020). Safety and efficacy of the BNT162b2 mRNA COVID-19 vaccine. N. Engl. J. Med..

[7] Skowronski D.M., De Serres G. (2021). Safety and Efficacy of the BNT162b2 mRNA COVID-19 Vaccine. N. Engl. J. Med..

[8] Xia X. (2021). Domains and functions of spike protein in SARS-CoV-2 in the context of vaccine design. Viruses.

[9] Huang Y., Yang C., Xu X.-F., Xu W., Liu S.-W. (2020). Structural and functional properties of SARS-CoV-2 spike protein: Potential antivirus drug development for COVID-19. Acta Pharmacol. Sin..

[10] Long Q.-X., Liu B.-Z., Deng H.-J., Wu G.-C., Deng K., Chen Y.-K., Liao P., Qiu J.-F., Lin Y., Cai X.-F. (2020). Antibody responses to SARS-CoV-2 in patients with COVID-19. Nat. Med..

[11] Angulo F.J., Finelli L., Swerdlow D.L. (2021). Estimation of US SARS-CoV-2 infections, symptomatic infections, hospitalizations, and deaths using seroprevalence surveys. JAMA Netw. Open.

[12] Ong D.S., Fragkou P.C., Schweitzer V.A., Chemaly R.F., Moschopoulos C.D., Skevaki C. (2021). How to interpret and use COVID-19 serology and immunology tests. Clin. Microbiol. Infect..

[13] Lai C.-C., Wang J.-H., Hsueh P.-R. (2020). Population-based seroprevalence surveys of anti-SARS-CoV-2 antibody: An up-to-date review. Int. J. Infect. Dis..

[14] Theel E.S., Slev P., Wheeler S., Couturier M.R., Wong S.J., Kadkhoda K. (2020). The role of antibody testing for SARS-CoV-2: Is there one?. J. Clin. Microbiol..

[15] Korth J., Wilde B., Dolff S., Anastasiou O.E., Krawczyk A., Jahn M., Cordes S., Ross B., Esser S., Lindemann M. (2020). SARS-CoV-2-specific antibody detection in healthcare workers in Germany with direct contact to COVID-19 patients. J. Clin. Virol..

[16] Pollán M., Pérez-Gómez B., Pastor-Barriuso R., Oteo J., Hernán M.A., Pérez-Olmeda M., Sanmartín J.L., Fernández-García A., Cruz I., de Larrea N.F. (2020). Prevalence of SARS-CoV-2 in Spain (ENE-COVID): A nationwide, population-based seroepidemiological study. Lancet.

[17] Nicol T., Lefeuvre C., Serri O., Pivert A., Joubaud F., Dubée V., Kouatchet A., Ducancelle A., Lunel-Fabiani F., Le Guillou-Guillemette H. (2020). Assessment of SARS-CoV-2 serological tests for the diagnosis of COVID-19 through the evaluation of three immunoassays: Two automated immunoassays (Euroimmun and Abbott) and one rapid lateral flow immunoassay (NG Biotech). J. Clin. Virol..

[18] Montesinos I., Dahma H., Wolff F., Dauby N., Dalunoy S., Wuyts M., Detemmerman C., Duterme C., Vandenberg O., Martin C. (2021). Neutralizing antibody responses following natural SARS-CoV-2 infection: Dynamics and correlation with commercial serologic tests. J. Clin. Virol..

[19] Wolff F., Dahma H., Duterme C., Van den Wijngaert S., Vandenberg O., Cotton F., Montesinos I. (2020). Monitoring antibody response following SARS-CoV-2 infection: Diagnostic efficiency of 4 automated immunoassays. Diagn. Microbiol. Infect. Dis..

[20] Montesinos I., Gruson D., Kabamba B., Dahma H., Van den Wijngaert S., Reza S., Carbone V., Vandenberg O., Gulbis B., Wolff F. (2020). Evaluation of two automated and three rapid lateral flow immunoassays for the detection of anti-SARS-CoV-2 antibodies. J. Clin. Virol..

[21] Galanis P., Vraka I., Fragkou D., Bilali A., Kaitelidou D. (2021). Seroprevalence of SARS-CoV-2 antibodies and associated factors in healthcare workers: A systematic review and meta-analysis. J. Hosp. Infect..

[22] Sahu A.K., Amrithanand V., Mathew R., Aggarwal P., Nayer J., Bhoi S. (2020). COVID-19 in health care workers—A systematic review and meta-analysis. Am. J. Emerg. Med..

[23] Lorent D., Nowak R., Roxo C., Lenartowicz E., Makarewicz A., Zaremba B., Nowak S., Kuszel Ł., Stefaniak J., Kierzek R. (2021). Prevalence of anti-SARS-CoV-2 antibodies in Poznań, Poland, after the first wave of the COVID-19 pandemic. Vaccines.

[24] Herzberg J., Vollmer T., Fischer B., Becher H., Becker A.-K., Honarpisheh H., Guraya S.Y., Strate T., Knabbe C. (2021). SARS-CoV-2-antibody response in health care workers after vaccination or natural infection in a longitudinal observational study. Vaccine.

[25] Sonmezer M.C., Erul E., Sahin T.K., Al I.R., Cosgun Y., Korukluoglu G., Zengin H., Dizman G.T., Inkaya A.C., Unal S. (2022). Seroprevalence of SARS-CoV-2 antibodies and associated factors in healthcare workers before the era of vaccination at a tertiary care hospital in Turkey. Vaccines.

[26] Houlihan C.F., Vora N., Byrne T., Lewer D., Kelly G., Heaney J., Gandhi S., Spyer M.J., Beale R., Cherepanov P. (2020). Pandemic peak SARS-CoV-2 infection and seroconversion rates in London frontline health-care workers. Lancet.

[27] Shields A., Faustini S.E., Perez-Toledo M., Jossi S., Aldera E., Allen J.D., Al-Taei S., Backhouse C., Bosworth A., Dunbar L.A. (2020). SARS-CoV-2 seroprevalence and asymptomatic viral carriage in healthcare workers: A cross-ectional study. Thorax.

[28] Kwiecińska-Piróg J., Przekwas J., Kraszewska Z., Sękowska A., Brodzka S., Wiktorczyk-Kapischke N., Grudlewska-Buda K., Wałecka-Zacharska E., Zacharski M., Mańkowska-Cyl A. (2021). The differences in the level of anti-SARS-CoV-2 antibodies after mRNA vaccine between convalescent and non-previously infected people disappear after the second dose—Study in healthcare workers group in Poland. Vaccines.

[29] Miyajima E., Imaizumi H., Oshida S., Igarashi K., Yoshida M., Yanase N. (2022). Survey of spike-specific immunoglobulin G antibodies at approximately 3 months and 9 months after vaccination against coronavirus disease 2019 (severe acute respiratory syndrome coronavirus-2 [SARS-CoV-2]) in health care workers. Sangyo Eiseigaku Zasshi.

[30] Bleier B.S., Ramanathan M., Lane A.P. (2021). COVID-19 vaccines may not prevent nasal SARS-CoV-2 infection and asymptomatic transmission. Otolaryngol. Head Neck Surg..

[31] Noda K., Matsuda K., Yagishita S., Maeda K., Akiyama Y., Terada-Hirashima Y., Matsushita H., Iwata S., Yamashita K., Atarashi Y. (2021). A novel highly quantitative and reproducible assay for the detection of anti-SARS-CoV-2 IgG and IgM antibodies. Sci. Rep..

[32] The U.S. Food and Drug Administration, 2021 Safety Communications (2021). Antibody Testing Is Not Currently Recommended to Assess Immunity after COVID-19 Vaccination: FDA Safety Communication. https://www.fda.gov/medical-devices/safety-communications/antibody-testing-not-currently-recommended-assess-immunity-after-COVID-19-vaccination-fda-safety.

[33] Padoan A., Dall’Olmo L., della Rocca F., Barbaro F., Cosma C., Basso D., Cattelan A., Cianci V., Plebani M. (2021). Antibody response to first and second dose of BNT162b2 in a cohort of characterized healthcare workers. Clin. Chim. Acta.

[34] Kumar N., Bhartiya S., Desai S., Mutha A., Beldar A., Singh T. (2021). Seroprevalence of antibodies against SARS-CoV-2 among health care workers in Mumbai, India. Asia Pac. J. Public Health.

[35] Zeng F., Dai C., Cai P., Wang J., Xu L., Li J., Hu G., Wang Z., Zheng F., Wang L. (2020). A comparison study of SARS-CoV-2 IgG antibody between male and female COVID-19 patients: A possible reason underlying different outcome between sex. J. Med. Virol..

[36] Wang W., Balfe P., Eyre D.W., Lumley S.F., O’Donnell D., Warren F., Crook D.W., Jeffery K., Matthews P.C., Klerman E.B. (2022). Time of day of vaccination affects SARS-CoV-2 antibody responses in an observational study of health care workers. J. Biol. Rhythm..

